# Clinical utility of the Mood and Anxiety Symptom Questionnaire (MASQ) in a sample of young help-seekers

**DOI:** 10.1186/1471-244X-7-50

**Published:** 2007-09-17

**Authors:** Joe A Buckby, Alison R Yung, Elizabeth M Cosgrave, Eoin J Killackey

**Affiliations:** 1 ORYGEN Research Centre, Melbourne, Australia; 2 University of Melbourne, Department of Psychiatry, Melbourne, Australia; 3 University of Melbourne, Department of Psychology, Melbourne, Australia

## Abstract

**Background:**

The overlap between Depression and Anxiety has led some researchers to conclude that they are manifestations of a broad, non-specific neurotic disorder. However, others believe that they can be distinguished despite sharing symptoms of general distress. The Tripartite Model of Affect proposes an anxiety-specific, a depression-specific and a shared symptoms factor. Watson and Clark developed the Mood and Anxiety Symptom Questionnaire (MASQ) to specifically measure these Tripartite constructs. Early research showed that the MASQ distinguished between dimensions of Depression and Anxiety in non-clinical samples. However, two recent studies have cautioned that the MASQ may show limited validity in clinical populations. The present study investigated the clinical utility of the MASQ in a clinical sample of adolescents and young adults.

**Methods:**

A total of 204 Young people consecutively referred to a specialist public mental health service in Melbourne, Australia were approached and 150 consented to participate. From this, 136 participants completed both a diagnostic interview and the MASQ.

**Results:**

The majority of the sample rated for an Axis-I disorder, with Mood and Anxiety disorders most prevalent. The disorder-specific scales of the MASQ significantly discriminated Anxiety (61.0%) and Mood Disorders (72.8%), however, the predictive accuracy for presence of Anxiety Disorders was very low (29.8%). From ROC analyses, a proposed cut-off of 76 was proposed for the depression scale to indicate 'caseness' for Mood Disorders. The resulting sensitivity/specificity was superior to that of the CES-D.

**Conclusion:**

It was concluded that the depression-specific scale of the MASQ showed good clinical utility, but that the anxiety-specific scale showed poor discriminant validity.

## Background

### The Tripartite model

Mood and anxiety disorders are often comorbid [1,2]. This finding is not surprising given the symptom overlap between these two syndromes [3]. A Tripartite model of Affect has been proposed to account for this overlap and to identify the unique components of anxiety and depression [4]. Under this model, Negative Affect (NA), a mixed anxiety-depression factor, explains the comorbidity between these syndromes. Positive Affect (PA) is the depression-specific factor and Physiological Hyperarousal (PH) is unique to anxiety.

There is some evidence for the Tripartite model for its ability to distinguish between depression and anxiety [5-9]. However, some findings have suggested the need to modify the original model [10]. For example, PH may only be related to Panic Disorder and, to a lesser extent, Generalised Anxiety Disorder.

Much of the research into the validity of the tripartite model has been conducted with measures that were not specifically designed to measure the tripartite constructs (e.g. BDI, BAI). Specific measures may be necessary to explore the utility of the model [11].

### The Mood and Anxiety Symptom Questionnaire

The Mood and Anxiety Symptom Questionnaire (MASQ) was designed to be a specific measure of the Tripartite model of Affect [12,13]. Three general distress scales measure the postulated shared factor, NA. A depression- (Anhedonic Depression, AD) and anxiety-specific (Anxious Arousal, AA) scale measures PA and PH respectively. Watson et al. reported good discriminant and convergent validity for the MASQ across five samples comprising college students, adults sampled from the community and patients presenting to a pain clinic. Factorial validity for the MASQ has been established in non-clinical samples, with three factors consistently found to best represent the data [7,13,14]. However, a recent study employed confirmatory factor analysis with depressed and anxious patients and reported that the structure of the tripartite model was not confirmed in this clinical sample [15]. Additionally, two recent studies have questioned the discriminant validity of the MASQ in clinical samples [16,17]. Discriminant validity reflects the extent to which an instrument measures the construct it is purported to measure, without inadvertently measuring other domains/constructs. Buckby et al. reported that depressed participants rated higher than non-depressed participants on all MASQ scales, including the anxiety-specific AA. Furthermore, they reported that participants with anxiety disorders did not score higher than participants without a current disorder on any scale. Boschen and Oei [16] reported that although the MASQ scales significantly discriminated between anxiety and depression, there was little clinical utility in these findings as the maximum discrimination was only 70%. Both studies cautioned future researchers to be mindful of the potential weakness of the MASQ's validity in clinical samples and indicated that further research, including replication, was necessary to better understand this issue.

### Rationale and hypotheses

Boschen & Oei [16] used a clinical sample of adults with Mood and Anxiety disorders. A recent review has criticised researchers for unduly focusing on psychometric characteristics and neglecting sensitivity [18]. Using similar analyses to Boschen & Oei, the present study therefore attempted to replicate their findings in a clinical sample of adolescents and young adults in order to determine how generalisable those findings were. People in the early stages of mental disorders may be especially receptive to treatment and may require more benign interventions than chronic populations [19]. It is therefore important to be able to validly recognise psychological distress in people at-risk of mental disorder. Should the MASQ be determined to validly detect depression and anxiety, it could prove to be a useful tool that is easily administered by professions including GPs and triage workers. It was hypothesised that the MASQ would show marginal to weak criterion validity in the present sample. A secondary aim was to compare the MASQ depression scale, AD, with the Center for Epidemiologic Studies Depression Scale (CES-D) [20]. The CES-D is a widely-used measure of depressive symptomatology that has been validated in both adult and adolescent samples [20,21]. However, some researchers have questioned its specificity to depression. For example, the CES-D has high reported correlations with anxiety measures and contains items that are not specific to depression. The present study sought to determine whether the MASQ offered an improvement to this earlier measure.

## Methods

Ethics approval for this study was given by the local ethics board, the Melbourne Health research and ethics committee.

### Participants

Participants were 150 people aged 15–24 years consecutively referred to ORYGEN Youth Health (OYH), a specialist youth public mental health service in Melbourne, Australia. Sources of referral included family, GPs, hospitals and school counsellors.

### Procedures

Between April and September, 2003, all young people who were referred to OYH with non-psychotic disorders were approached by trained research interviewers. Consenting participants completed the MASQ and a diagnostic interview. Inter-rater assessments were conducted in approximately 15% of the cases to ensure agreement between interviewers. Kappa values for mood (0.89) and anxiety diagnoses (0.80) were high.

The sample, setting and methodology have been described in greater detail elsewhere [22,23].

### Measures

#### Mood and Anxiety Symptom Questionnaire (MASQ)

The MASQ is a 77-item self-report questionnaire that assesses depressive, anxious and mixed symptomatology [12,13]. Three scales measure General Distress: depressive symptoms (12 items), anxious symptoms (11 items) and mixed symptoms (15 items). There is also an anxiety-specific (Anxious Arousal, 17 items) and depression-specific scale (Anhedonic Depression, 22 items). Higher scores reflect greater levels of symptomatology. The reported internal consistency for each scale is excellent with coefficient alphas ranging from 0.78 to 0.92.

#### Structured Clinical Interview for DSM-IV (SCID-IV)

The SCID-IV was administered to assess for Axis-I disorders and is a semi-structured interview [24]. All assessments were conducted by trained research interviewers. Cases were presented at weekly clinical meetings with experienced doctoral-level clinical psychologists confirming diagnoses.

#### Centre for Epidemiologic Studies- Depression Scale (CES-D)

The CES-D is a 20-item self-report questionnaire that assesses depressive symptomatology [20]. The psychometric properties of the CES-D have been established in both adult and adolescent samples [20,21]. Higher CES-D scores reflect greater levels of symptomatology.

## Results

### Characteristics of the sample

Two hundred and four people met the study criteria during the recruiting phase. Of the 150 consenting participants 14 did not return valid questionnaires, leaving a sample of 136 with useable data. There was no significant demographic differences between those with, and without missing data. A small proportion of participants (< 10%) had several, random missing variables replaced with the Expectation Maximisation method [25].

The mean age of participants was 18.11 years (SD = 2.61) and the sample was comprised of 61% females (N = 83). There was no significant gender difference in MASQ scores. There was a significant difference between adolescents (age 15–17) and young adults (age 18–24) on all MASQ scales except AA with older participants scoring higher.

The majority of the sample (80.1%, N = 109) rated for a current Axis-I disorder. Mood and Anxiety disorders were most common. The sample was split according to diagnostic status: Mood Disorder only (no comorbid anxiety, N = 29, 21.3%); Anxiety Disorder only (no comorbid mood, n = 22, 16.2%); Comorbid Anxiety-Mood (n = 35, 25.7%); and no Anxiety or Depression (n = 50, 36.8%). The most common Anxiety disorders were specific phobia (16.9%), social phobia (15.4%) and panic disorder (11.0%). Post traumatic stress (8.1%), generalised anxiety (5.1%) and obsessive compulsive (6.6%) disorders were less frequent. Nearly half (46%) of the no anxiety-mood group rated for another Axis-I diagnosis (including substance abuse/dependence and eating disorders). Prevalence of other disorders has been described elsewhere [2].

### Descriptive statistics and correlational analyses

Means and standard deviations are presented in Table 1. Inter-correlations between all MASQ scales were uniformly high and statistically significant with the lowest correlation between the disorder-specific scales AA and AD (r = 0.59, see Table 2). Inter-correlations between AA:AD were inspected across the diagnostic groups. This correlation was high in all groups rating for either a Mood or Anxiety Disorder with the highest correlation in Anxiety only (r = 0.62), followed by Comorbid (r = 0.60) and Mood only (r = 0.46) (p < .001 for all). In contrast, AA:AD was only moderately correlated in the no Anxiety/Mood group (r = 0.35, p = 0.01). When examining those participants without any current diagnoses (N = 27), AA:AD were uncorrelated (r = 0.18, p = 0.36). This finding indicated a differential relationship for the correlation between those with (r = 0.59, p < .001, N = 109) and those without an Axis-I disorder. Therefore, an independent samples t-test of the correlational coefficients was conducted [26], showing a significantly higher correlation in a composite diagnosis group that included participants with any current disorder (z = 4.04, p < .001).

**Table 1:** Descriptive statistics for MASQ and CES-D scores

	Total sample (N = 136)	Mood only (N = 29)	Anxiety only (N = 22)	Comorbid (N = 35)	No Anxiety/Depression (N = 50)
	Mean (SD)				

GD: M	44.20 (14.40)	49.04 (14.98)	37.55 (13.45)	53.20 (11.37)	38.01 (12.06)
GD: A	25.17 (9.40)	28.80 (9.27)	21.82 (7.73)	31.39 (9.46)	20.17 (6.40)
GD: D	33.31 (12.86)	37.63 (12.78)	27.72 (11.88)	42.77 (10.78)	26.63 (9.31)
AA	34.08 (13.38)	38.11 (16.38)	30.63 (11.18)	40.94 (13.26)	28.46 (9.14)
AD	75.81 (17.43)	82.82 (8.31)	67.18 (17.13)	87.80 (12.24)	67.13 (14.05)
CES-D	26.71 (9.75)	30.69 (8.31)	22.05 (9.23)	32.66 (9.78)	22.20 (7.35)

*Note*: GD: M = General Distress: Mixed; GD: A = General Distress: Anxious; GD: D = General Distress: Depressive; AA = Anxious Arousal; AD = Anhedonic Depression.

**Table 2:** Inter-correlations between MASQ scales and CES-D

	GD: M	GD: A	GD: D	AA	AD	CES-D
GD: M	[0.92]					
GD: A	0.86*	[0.88]				
GD: D	0.86*	0.83*	[0.93]			
AA	0.74*	0.80*	0.69*	[0.91]		
AD	0.79*	0.75*	0.82*	0.59*	[0.93]	
CES-D	0.82*	0.79*	0.83*	0.65*	0.72*	[0.93]

*Note*: Figures on the diagonal in parentheses denote Cronbach's alpha, a measure of internal consistency.

* denotes p < .001.

### Logistic regression analyses- Predicting mood disorder

To replicate earlier findings [16] logistic regression analyses were run with diagnostic status as the dependent variable. In the first analysis, a dichotomous outcome variable was created reflecting presence (n = 64, 47.1%) versus absence (n = 72) of a Mood Disorder. The depression-specific scale AD was included in the first block of covariates, the three GD scales in the second block and AA in the third block.

AD significantly predicted mood disorder (χ^2 ^(1) = 44.06, p < .001). The addition of the GD scales in the second block did not significantly increase the prediction of mood disorder (p = 0.09), nor did inclusion of AA at the third step (p = 0.78). The overall predictive accuracy of AD for a mood disorder was 72.8% (71.9% for presence and 73.6% for absence of a mood disorder).

### Logistic regression analyses- Predicting anxiety disorder

In the second analysis, presence (n = 57, 41.9%) versus absence (n = 79) of an anxiety disorder was the dependent variable. The anxiety-specific scale AA was included in the first step, the GD scales in the second, and AD in the third step.

AA significantly predicted anxiety disorder (χ^2 ^(1) = 4.58, p = 0.03). The GD scales did not increase the prediction (p = 0.15), nor did AD in the final step (p = 0.79). The overall predictive accuracy of AA for an anxiety disorder was 61.0% (29.8% for presence and 83.5% for absence).

### ROC analyses

The ROC curve allows researchers to graphically determine a test's sensitivity and specificity [27]. The area under the curve of a ROC plot provides a score that ranges from 1 (test is always correct) to 0 (test is never correct). A score of 0.5 indicates that the result is no better than chance. When comparing different tests, the one with the larger area under the curve is determined to be the better diagnostic tool.

In the first analysis, the dichotomous Mood disorder variable was entered as the state variable and the disorder-specific scales AA and AD and the CES-D were entered as the test variables. The ROC plot for AD contained 81.8% under the curve (SE = 0.04, 95% C.I. = 0.75, 0.89), AA contained 72.3% (SE = 0.04, 95% C.I. = 0.64, 0.81) and CES-D contained 78.7% (SE = 0.04, 95% C.I. = 0.71, 0.86).

In the second analysis, the dichotomous anxiety variable was input as the state variable and the disorder-specific scales as the test variables. The ROC plot for AA contained 61.7% under the curve (SE = 0.05, 95% C.I. = 0.52, 0.72), AD contained 62.0% (SE = 0.05, 95% C.I. = 0.53, 0.72) and CES-D contained 58.3% (SE = 0.05, 95% C.I. = 0.48, 0.68).

**Figure 1:**
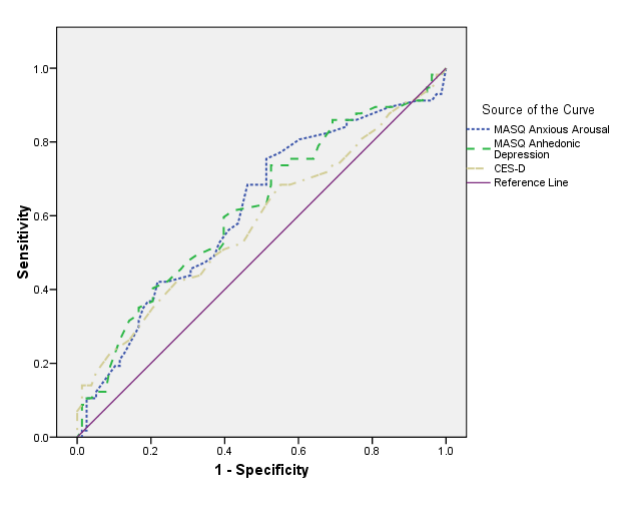
ROC analyses for Anxiety diagnosis by AA, AD and CES-D.

**Figure 2:**
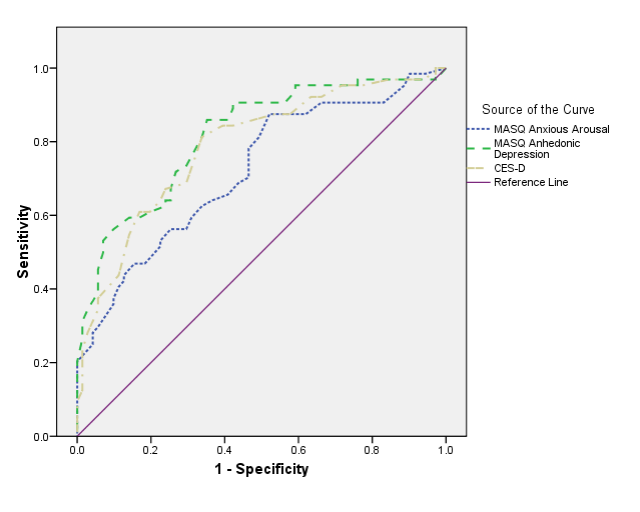
ROC analyses for Mood diagnosis by AA, AD and CES-D.

The sensitivity and specificity of AD was further inspected to identify a potential cut-off to indicate 'caseness' for depression. By visually inspecting the ROC for the largest area under the curve and by manually comparing various true positive and true negative rates, it was determined that an AD cut-off of 76 best reflected caseness (sensitivity = 85%, specificity = 65%). These figures were then compared to CES-D (cut-off = 24). AD is equivalent to, if not better than, the CES-D (sensitivity = 84%, specificity = 61%) in discriminating Mood Disorders when utilising the proposed cut-off of 76. Further analyses with AA were not run as this scale demonstrated poor discriminant validity (see above) (Figures 1 and 2).

## Discussion

The current study investigated the ability of the MASQ's AD and AA scales to distinguish between Anxiety and Mood Disorders. We sought to assess the discriminant validity of the MASQ in a clinical sample of adolescents and young adults (mean age = 18.11 years). We attempted to replicate and extend statistical methods used by Boschen & Oei [16], who had a sample of 470 adult patients with Mood and Anxiety Disorders (mean age = 34.30 years). Present results largely supported the earlier findings.

First, the disorder-specific scales AA and AD were significant predictors of the target dependent variable anxiety and depression respectively. AD was superior to AA in predicting its target syndrome. AD accurately predicted Mood disorders (72.8%). However, AA weakly predicted Anxiety disorders (61% of overall cases). Interestingly, AA predicted the absence of anxiety well (83.5%) but very poorly predicted the presence of these disorders (29.8%). These findings add further support to earlier analyses with the same sample [17]. In that study we reported that depressed participants scored highly on all scales but that those rating for Anxiety Disorders were not distinguished from those without an Axis-I disorder. The present findings are comparable to those obtained by Boschen & Oei, who reported depression being predicted 66.4% and anxiety 61.1% of the time. There are some notable differences between the present results and those of Boschen & Oei. First, the slightly higher prediction of depression in the present study is related to a more accurate prediction of the presence of depressive disorder by AD in our sample. In Boschen & Oei's study, the presence of depression was only predicted 42.3% of the time (versus 71.9% in the current study). Second, though the overall prediction of Anxiety is identical across studies, very low prediction of Anxiety Disorder was reported in the current study but was high (81.5%) in Boschen & Oei's study. Third, the three MASQ general distress scales did not significantly increase the prediction above that explained by the disorder-specific scales in this replication. They were significant predictors in the original study.

The ROC analyses, were also supportive of the earlier findings reported by Boschen & Oei [16], particularly for the weak utility of the AA scale. Results were more positive for AD. When implementing the proposed cut-off for the AD scale (76 or greater), 85% of depressed participants were correctly identified. The low true negative rate (non-depressed participants incorrectly identified, 65%) is comparable to that reported elsewhere with adolescent samples [28]. As we have argued elsewhere [17], anxiety and depressive syndromes may not yet be clearly distinguished in populations similar to the present sample, which may account for the MASQ's failure to distinguish between them.

Results from the present correlational analyses further illustrate the psychometric limitations of the MASQ in the present sample. Though specifically designed to distinguish distinct depressive, anxious and general distress syndromes, all five MASQ scales were highly inter-correlated. The AA:AD correlation was lowest, but still highly and significantly correlated in all participants rating for a current Axis-I disorder (r = 0.59). This correlation is particularly high for scales that are supposedly separate constructs and provides further evidence for this measure's limitations in clinical samples. A closer inspection of this correlation in those participants without a current disorder revealed these disorder-specific scales were not correlated (r = 0.18). These results provide further evidence that the MASQ, and hence the tripartite model, will require significant revision for use with clinical samples. The results presented in the current study suggest that that anxiety-specific scale, AA, may be most in need of revision.

The present results provide further support for the notion that caution be employed by researchers when planning the use of the MASQ in clinical samples. All scales are highly correlated, limiting the utility of the scale for distinguishing between Anxiety and Mood Disorders. Results from both logistic regression and ROC analyses showed that the depression-specific scale, AD, had good discriminant validity in detecting the presence of Mood Disorders and also demonstrated clinically useful sensitivity and specificity. The anxiety-specific scale, AA, however, showed poor clinical utility in the present sample. This may be because the anxiety construct of the tripartite model, PH, has been found to have heterogeneous relationship with Anxiety Disorders. Only Panic Disorder and, to a lesser extent, Generalised Anxiety Disorder have been established to relate to PH [10]. Obsessive Compulsive Disorder and Social Phobia may be unrelated. Very few participants in the present sample rated for GAD or Panic Disorder (11.0 and 5.1% respectively), therefore it is not surprising that the AA scale did not demonstrate clinical utility. Further studies are necessary to elucidate this issue to better determine the settings under which the MASQ is most valid. In support of the current results, a Dutch translation of the MASQ found only limited uniqueness of the anxious-specific scale (compared to Negative Affect) [29]. It may be that future revisions of the AA scale will require the addition of items that are representative of the spectrum of anxiety disorders. The inclusion of items that relate to phobic avoidance and/or worrying may enhance the clinical utility of this scale. At present, AA may be considered to assess primarily panic symptoms. Replication of current methods would also be beneficial in similar samples to establish the generalisability of the present findings.

Though Boschen & Oei [16] concluded that both MASQ specific scales demonstrated weak clinical utility, we argue that the predictive utility of the AD scale (73%) may be high enough for clinical and research use. Though the MASQ was not designed for this purpose, the AD scale may be superior to the widely-used CES-D in determining clinical 'caseness' for Mood Disorders. A brief (22 item), cheap self-report screening tool that accurately discriminates three out of four people who may warrant specialist clinical services may be well-received by mental health triage workers. Though the present sample is not large, this is the first study that the authors are aware of that has attempted to define a clinical cut-off for a MASQ scale. Present results indicate that AD is slightly superior to the CES-D in the current sample.

## Competing interests

The author(s) declare that they have no competing interests.

## Authors' contributions

JB collected the data, performed the statistical analyses, formulated the research question and wrote this manuscript. AY conceived the study and participated in its design and helped draft the manuscript. EC participated in the design of the study and co-ordination and helped to draft the manuscript. EK participated in the design of the study and co-ordination. All authors read and approved the final manuscript.

## Pre-publication history

The pre-publication history for this paper can be accessed here:


